# Phenotypic Identification of the Redox Dye Methylene Blue as an Antagonist of Heat Shock Response Gene Expression in Metastatic Melanoma Cells

**DOI:** 10.3390/ijms14024185

**Published:** 2013-02-19

**Authors:** Angela L. Davis, Christopher M. Cabello, Shuxi Qiao, Sara Azimian, Georg T. Wondrak

**Affiliations:** Pharmacology and Toxicology, College of Pharmacy, Arizona Cancer Center, University of Arizona, Tucson, AZ 85724, USA; E-Mails: davis@pharmacy.arizona.edu (A.L.D.); cabello@pharmacy.arizona.edu (C.M.C.); qiao@pharmacy.arizona.edu (S.Q.); azimian@pharmacy.arizona.edu (S.A.)

**Keywords:** malignant melanoma, heat shock response, Hsp70, Hsp27, methylene blue, chemosensitization

## Abstract

Repurposing approved and abandoned non-oncological drugs is an alternative developmental strategy for the identification of anticancer therapeutics that has recently attracted considerable attention. Due to the essential role of the cellular heat shock response in cytoprotection through the maintenance of proteostasis and suppression of apoptosis, small molecule heat shock response antagonists can be harnessed for targeted induction of cytotoxic effects in cancer cells. Guided by gene expression array analysis and a phenotypic screen interrogating a collection of 3,7-diamino-phenothiazinium derivatives, we have identified the redox-drug methylene blue (MB), used clinically for the infusional treatment of methemoglobinemia, as a negative modulator of heat shock response gene expression in human metastatic melanoma cells. MB-treatment blocked thermal (43 °C) and pharmacological (celastrol, geldanamycin) induction of heat shock response gene expression, suppressing Hsp70 (*HSPA1A*) and Hsp27 (*HSPB1*) upregulation at the mRNA and protein level. MB sensitized melanoma cells to the apoptogenic activity of geldanamycin, an Hsp90 antagonist known to induce the counter-regulatory upregulation of Hsp70 expression underlying cancer cell resistance to geldanamycin chemotherapy. Similarly, MB-cotreatment sensitized melanoma cells to other chemotherapeutics (etoposide, doxorubicin). Taken together, these data suggest feasibility of repurposing the non-oncological redox drug MB as a therapeutic heat shock response antagonist for cancer cell chemosensitization.

## 1. Introduction

When exposed to cytotoxic stress, mammalian cells activate a number of conserved cytoprotective molecular pathways including the heat shock response, the ER stress/unfolded protein response, and the oxidative stress response [1–3]. The cellular heat shock response is mediated by heat shock proteins (Hsps), cytoprotective factors encoded by evolutionarily conserved gene families controlled by specialized stress-responsive transcription factors (termed heat shock factors). Hsps are involved in protein folding and control of protein aggregation, complex formation, transport and degradation, processes jointly termed “proteostasis” [3]. Specific heat shock proteins including members of the Hsp27, Hsp70, and Hsp90 families are overexpressed and/or functionally altered in cancer cells contributing to increased survival, stress resistance, and chemoresistance [4–6].

Due to the essential role of the cellular heat shock response in cytoprotection, proteostasis, and suppression of apoptosis, small molecule heat shock response antagonists can be harnessed for the targeted induction of cytotoxic effects in cancer cells [4–10]. For example, the cytosolic chaperone heat shock protein 90 (Hsp90) represents a promising molecular target for chemotherapeutic intervention due to its causative involvement in the stabilization of mutant client proteins and facilitation of oncogenic signaling cascades, and ansamycin-based and non-ansamycin-based small molecule inhibitors of Hsp90 are currently under clinical evaluation in human cancer patients [11,12]. In the Hsp70 family, the stress-inducible heat shock protein Hsp70 (also called Hsp72 or Hsp70-1, encoded by *HSPA1A*), an ATP-dependent chaperone overexpressed in many tumor types, has emerged as a causative factor in tumorigenesis and is now considered a high quality target for therapeutic intervention aiming at cancer cell chemosensitization [5,10]. Genetic depletion and small molecule antagonism of Hsp70 have been shown to cause chemosensitization of cancer cells [4,7,10].

Melanoma, a malignant tumor derived from melanocytes, causes the majority of deaths attributed to skin cancer, even though recent progress in the design of melanoma-targeted therapies such as the V600E-mutation directed BRAF-inhibitor vemurafenib has been achieved [13,14]. Efficacy of chemotherapeutic intervention directed against the metastatic stage of the disease remains limited, creating an urgent need for the identification and development of improved antimelanoma agents. Pathological alterations affecting expression and function of heat shock proteins (including Hsp27, Hsp70, and GRP78) have been observed in human melanoma tissue and are thought to contribute to the notorious chemoresistance of metastatic melanoma cells [15–23].

Recently, in an attempt to identify small molecule redox therapeutics for experimental antimelanoma intervention, we have demonstrated the melanoma cell-directed cytotoxicity of 3,7-diamino-phenothiazinium drugs focusing on the apoptogenic activity of toluidine blue in G361 metastatic melanoma cells [2,24,25]. Guided by follow up gene expression array analysis and phenotypic screening of various 3,7-diaminophenothiazinium-derivatives, we now report for the first time that the redox dye methylene blue (MB; 3,7-bis(dimethylamino)-phenothiazin-5-ium chloride; CAS#: 61-73-4) displays significant activity as a functional antagonist of heat shock response gene expression in cultured human metastatic melanoma cells. We demonstrate that MB-treatment attenuates thermally- and pharmacologically-induced heat shock response gene expression, suppressing Hsp70 (*HSPA1A*) and Hsp27 (*HSPB1*) upregulation at the mRNA and protein level, and causing chemosensitization of melanoma cells to geldanamycin cytotoxicity.

Currently, MB serves as a redox therapeutic clinically used for the infusional treatment of methemoglobinemia and widely studied as an investigational oral antimalarial [26,27]. As an inhibitor of nitric oxide synthase and guanylate cyclase, hemodynamic activities of methylene blue have been employed clinically in the context of postoperative vasoplegia, cardiac surgery, and septic shock [28–30]. Moreover, MB is currently undergoing human clinical trials for the treatment of cognitive disorders in the context of neurodegenerative diseases, a potential application that has been attributed to multiple activities such as inhibition of tau protein aggregation and modulation of cellular energy metabolism as an alternative electron carrier in the mitochondrial respiratory chain [31–38]. Here, we present prototype data that suggest feasibility of repurposing MB as an investigational drug for the attenuation of heat shock response gene expression causing chemosensitization of human metastatic melanoma cells.

## 2. Results and Discussion

### 2.1. Downregulation of Heat Shock Response Gene Expression in Human A375 and G361 Metastatic Melanoma Cells Exposed to the Redox-Active Phenothiazine-Derivative Methylene Blue

Recently, we have reported the redox-dependent apoptogenic activity of phenothiazine dyes targeting cultured melanoma cells identifying toluidine blue as an experimental redox chemotherapeutic [24]. Among the phenothiazine-derivatives tested originally, methylene blue (MB) displayed negligible apoptogenicity while causing pronounced oxidative stress in melanoma cells.

**Figure 1:**
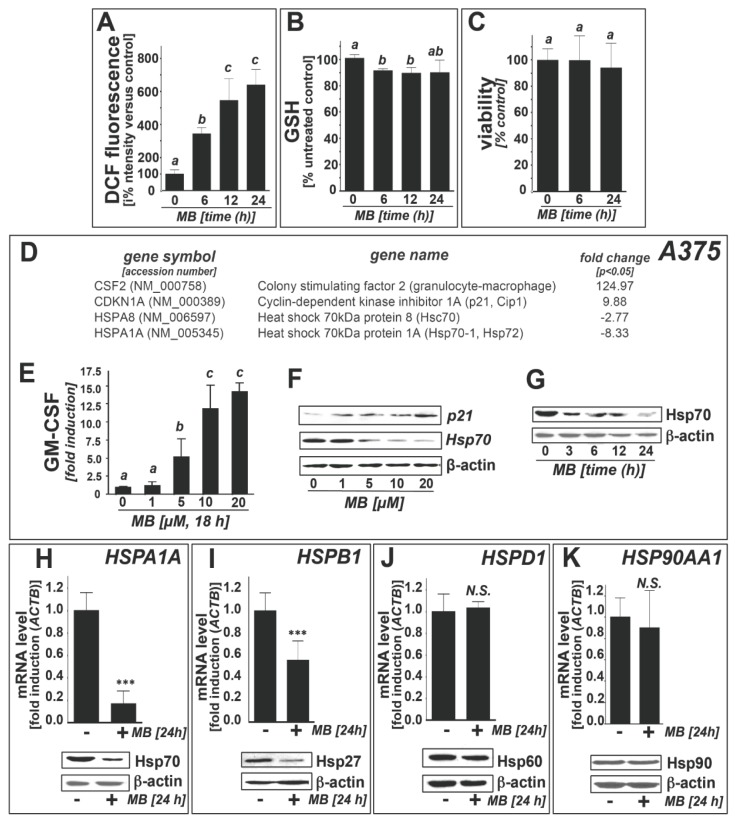
Gene expression array analysis reveals heat shock response downregulation in human A375 melanoma cells exposed to methylene blue. (**a**) methylene blue (MB)-induction of cellular oxidative stress as monitored by DCF fluorescence (flow cytometric analysis; MB, 10 μM, ≤24 h exposure); (**b**) Depletion of reduced cellular glutathione; (**c**) Cell viability was examined using flow cytometric analysis (annexin V-PI staining). Numbers in the bar graph indicate viable (AV-negative, PI-negative) in percent of total gated cells (mean ± SD, *n* = 3); (**d**) gene expression changes by at least twofold (*p* < 0.05; RT^2^ Human Stress and Toxicity Profiler™ PCR Expression Array technology; MB: 10 μM, 24 h) in A375 melanoma cells. Arrays were performed in three independent repeats and analyzed using the two-sided Student’s *t* test; (**e**) MB-modulation (1–20 μM, 18 h; *n* = 3) of GM-CSF2 protein levels in A375 cell culture medium as assessed by ELISA; (**f**) MB-modulation (1–20 μM, 24 h) of Hsp70 and p21 expression (immunoblot analysis); (**g**) MB-modulation (10 μM, 3–24 h) of Hsp70 protein expression (immunoblot analysis); (**h**–**k**) Heat shock response gene expression changes (*HSPA1A*, *HSPB1*, *HSPD1*, *HSP90AA1*) at the mRNA and protein level induced by MB-treatment (10 μM, 24 h) in A375 cells using independent quantitative RT-PCR (upper panels) and immunoblot analysis (lower panels).

To further explore the potential anti-melanoma activity of MB, we first assessed induction of oxidative stress that occurred in the absence of apoptotic effects (Figure 1c), measuring DCF fluorescence (indicative of the generation of oxidizing species) and glutathione depletion in A375 human metastatic melanoma cells exposed to MB (Figure 1a,b) [39]. MB-induction of significant intracellular oxidative stress was observed within 6 h exposure time, maintained over the 24 h duration of the experiment. As an independent marker of oxidative stress, we examined the occurrence of MB-induced glutathione depletion. In A375 cells, moderate reduction of reduced glutathione levels (approximately 10% of untreated control) was detectable within 6 h (10 μM; Figure 1b) suggesting that MB exposure, while not impairing melanoma cell viability over the extended observation period (24 h; Figure 1c), is associated with the induction of pronounced cellar redox dysregulation.

In an attempt to further characterize the complex stress response elicited by MB treatment at non-lethal concentrations, we performed focused expression array analysis. First, MB-induced modulation of stress and toxicity response gene expression was examined in A375 melanoma cells using the RT^2^ Human Stress and Toxicity Profiler™ PCR Expression Array technology (SuperArray, Frederick, MD, USA; MB: 10 μM, 24 h; Figure 1d) [25,39,40]. Out of 84 stress-related genes contained on the array MB-induced expression changes in A375 cells affected 4 genes (*CSF2*, *CDKN1A*, *HSPA8*, *HSPA1A*) by at least three-fold over untreated control cells. Massive upregulation of expression occurred with *CSF2*, the gene encoding granulocyte-macrophage colony stimulating factor (GM-CSF; approximately 125-fold), and *CDKN1A*, the stress-responsive tumor suppressor gene encoding cyclin-dependent kinase inhibitor 1A (p21; approximately 10 fold) [40].

MB-induced upregulation of GM-CSF in human melanoma cells may be of chemotherapeutic significance, since it has been shown recently that GM-CSF may be an effective agent for immunostimulatory antimelanoma intervention, an area of investigation beyond the scope of this study [41,42]. Importantly, significant downregulation of *HSPA8* (encoding the constitutively expressed heat shock 70 kDa protein 8 (Hsc70, also referred to as heat shock cognate 71 kDa protein or Hsp73); almost three-fold) and *HSPA1A* (encoding the inducible heat shock 70 kDa protein 1A (Hsp70, also called Hsp72 or Hsp70-1); approximately eight-fold) was observed [5].

Next, MB-induced gene expression changes were examined at the protein level (Figure 1e,g). ELISA-based detection revealed an almost 15-fold increase in GM-CSF levels in the medium of A375 cells exposed to MB (10 μM, 24 h; Figure 1e), an effect also observable at lower micromolar concentrations (5 μM; data not shown). Immunoblot analysis also revealed MB-induced upregulation of p21 and pronounced downregulation of inducible Hsp70 protein levels that occurred at low micromolar concentrations (≥5 μM; Figure 1b). Consistent with upregulation of the cyclin dependent kinase inhibitor p21, MB displayed pronounced antiproliferative activity on A375 melanoma cells (IC_50_ = 660 ± 48 nM; data not shown). Next, time course analysis (0–24 h; 10 μM) of MB-induced Hsp70 modulation revealed that MB-induced downregulation is detectable at the protein level as early as within 3 h exposure time (Figure 1g).

We also examined MB-induced effects on the expression of other heat shock response genes (mRNA and protein level) using independent real time RT-PCR and immunoblot analysis (Figure 1h,k). Indeed, moderate downregulation of the small heat shock protein Hsp27 (encoded by *HSPB1*) was observed at the mRNA (approximately twofold) and protein level (Figure 1i). In contrast, expression of Hsp60 and Hsp90 (encoded by *HSPD1* and *HSP90AA1*, respectively) remained unaffected by MB (Figure 1j,k), both at the mRNA and protein level, indicating that MB-induced modulation of the cellular heat shock response causes differential effects on inducible Hsp70 (Hsp72) and Hsp27 (downregulation at the protein and mRNA level) *versus* Hsp60 and Hsp90 (no changes at the protein and mRNA level).

In the context of MB-induced attenuation of heat shock response gene expression as observed by us for the first time in cultured mammalian cells, it should be mentioned that an earlier report has demonstrated the direct inhibitory activity of MB on Hsp70-ATPase enzymatic activity assessed in a cell free biochemical assay; moreover, it has been shown that inactivation by MB may involve covalent cysteine modification of Hsp72 (encoded by *HSPA1A*) but not Hsc70 (encoded by *HSPA8*) [43–45]. Indeed, direct biochemical inactivation of Hsp70 subtypes by MB may represent an additional mechanism of MB-dependent heat shock response modulation operative in addition to the early, more global downregulation of Hsp70 (*HSPA1A*) and Hsp27 (*HSPB1*) expression as observed by us at the transcriptional and protein level in melanoma cells, a hypothesis to be tested by future experimentation.

**Figure 2:**
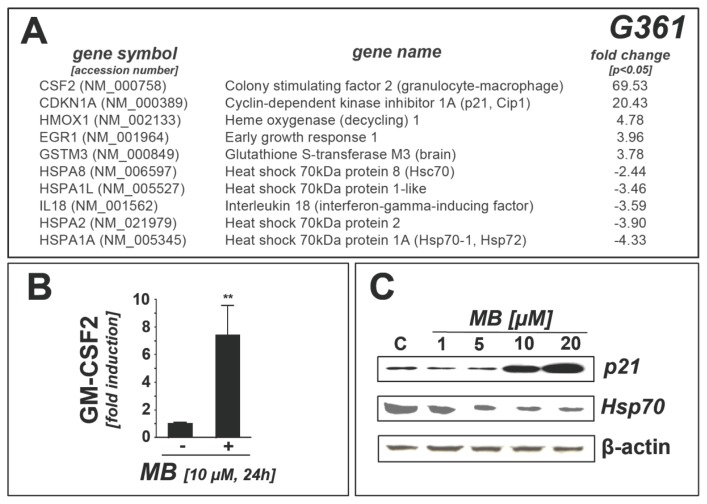
Gene expression array analysis indicates heat shock response downregulation in methylene blue-treated human G361 metastatic melanoma cells. (**a**) The table summarizes gene expression changes by at least twofold (*p* < 0.05) as detected by the RT^2^ Human Stress and Toxicity Profiler™ PCR Expression Array technology (MB: 10 μM, 24 h) in G361 melanoma cells. Arrays were performed in three independent repeats and analyzed using the two-sided Student’s *t* test; (**b**) MB-modulation (10 μM, 24 h) of GM-CSF2 protein levels in G361 cell culture medium as assessed by ELISA and analyzed using the Student’s *t* test; (**c**) MB-modulation (1–20 μM, 24 h) of Hsp70 and p21 protein expression as assessed by immunoblot analysis.

Importantly, key molecular changes observed in A375 melanoma cells in response to MB exposure were also reproduced in other human melanoma cell lines including G361 (amelanotic metastatic melanoma) (Figure 2). PCR expression array analysis revealed massive upregulation of *CSF2* (approximately 70 fold), confirmed at the protein level by detection of upregulated GM-CSF concentrations in cell culture medium using ELISA analysis (Figure 2b). Equally, as documented in MB-exposed A375 cells, pronounced upregulation of *CDKN1A* occurred at the mRNA (Figure 2a) and protein (p21) level (Figure 2c). Moreover, significant upregulation of oxidative stress-responsive genes (*HMOX1*, *GSTM3*, *EGR1*) was observed in MB-exposed G361 melanoma cells. Importantly, significant downregulation of heat shock response protein encoding genes (*HSPA1A*, *HSPA2*, *HSPA1L*, *HSPA8*) was observable at the mRNA level (Figure 2a), paralleled by downregulation of Hsp70 (Hsp72) protein levels observed by us upon exposure to MB concentrations as low as 5 μM (Figure 2c).

### 2.2. Modulation of Melanoma Cell Viability and Hsp70 Expression by MB-Related Phenothiazine-Derivatives

**Figure 3:**
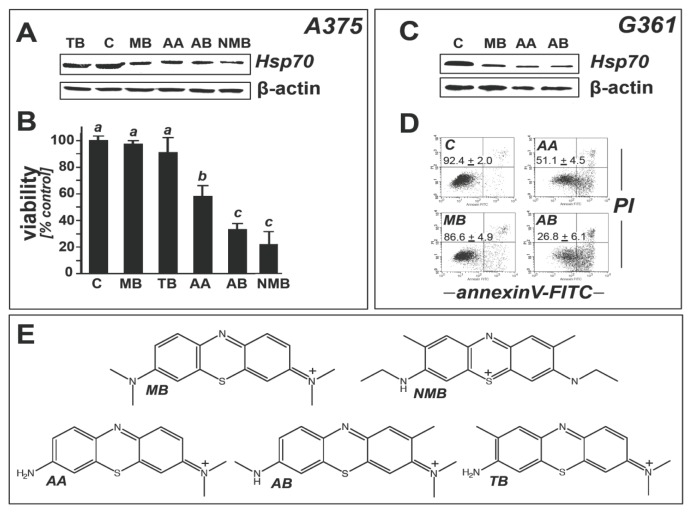
Comparative activity of phenothiazine-derivatives on melanoma cell viability and Hsp70 expression. (**A**) Hsp70 modulation as assessed by immunoblot analysis in A375 melanoma cells exposed to phenothiazine derivatives (10 μM, 24 h; methylene blue (MB), toluidine blue (TB), azure A (AA), azure B (AB), “new methylene blue” (NMB)) as assessed by immunoblot analysis. β-actin: loading control; (**B**) Viability analysis by flow cytometric analysis (all test compounds as used in (**A**)); (**C**) Hsp70 modulation as assessed by immunoblot analysis in G361 melanoma cells exposed to phenothioazine derivatives as specified in (**A**); (**D**) Viability analysis of G361 melanoma cells by flow cytometric analysis showing annexinV-PI panels; test compounds as used in (**A**); The numbers indicate viable (AV-negative, PI-negative) in percent of total gated cells (mean ± SD, *n* = 3). (**E**) Molecular structures of phenothiazine test compounds; abbreviations as in (**A**).

Next, a comparative assessment of Hsp70 modulation by MB-related 3,7-bis(dimethylamino)-phenothiazin-5-ium derivatives (chemical structures shown in Figure 3e) was performed using immunoblot analysis (Figure 3).

Among phenothiazine-derivatives we selected the MB-derived metabolites azure A (AA) and azure B (AB), formed physiologically from MB via hepatic desmethylation, and other closely related 3,7-bis(dimethylamino)-phenothiazin-5-ium derivatives including “new methylene blue” (NMB) and toluidine blue (TB) [46]. We observed that treatment with a number of MB-derivatives (AA, AB, NMB; 10 μM, 24 h) caused pronounced downregulation of Hsp70 expression in A375 (Figure 3a) and G361 melanoma cells (Figure 3c). In contrast, exposure to TB did not cause significant modulation of Hsp70 expression (Figure 3a), but induced *CSF2* mRNA upregulation as observed earlier with MB (data not shown). Viability analysis revealed potent apoptogenic effects associated with AA, AB, and NMB exposure (10 μM, 24 h), observed in A375 (Figure 3b) and G361 melanoma cells (Figure 3d).

Among all test compounds only MB displayed Hsp70-directed modulatory activity that occurred in the absence of induction of cell death. In order to avoid apoptogenicity as a confounding factor, for subsequent experimentation on heat shock response modulation and melanoma cell chemosensitization we therefore selected MB as our prototype phenothiazine-derived Hsp70 modulator. The mechanistic basis underlying MB-dependent heat shock response modulation and the potential involvement of MB-induced cellular oxidative stress as demonstrated in Figure 1a,b remains unknown at this point. Likewise, it remains to be seen if the ability of various MB-derivatives (AA, AB, NMB) to cause heat shock response modulation together with induction of apoptosis may be of potential chemotherapeutic use, but the underlying structure-activity relationship is unresolved at this point.

### 2.3. Methylene Blue Attenuates Thermal and Pharmacological Induction of Heat Shock Response Gene Expression

**Figure 4:**
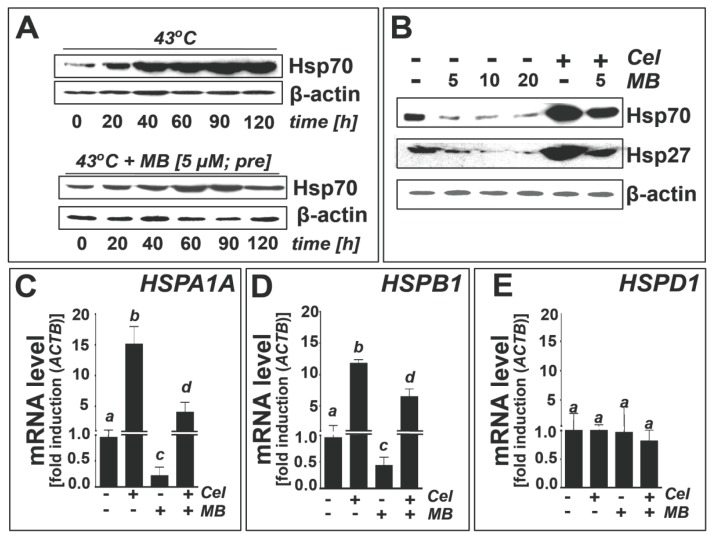
Methylene blue attenuates thermal and pharmacological induction of heat shock response gene expression in human A375 metastatic melanoma cells. (**a**) MB (10 μM, 1 h pretreatment) effects on heat-induced (43 °C, 0–120 min exposure followed by 6 h recovery) upregulation of Hsp70 protein levels (immunoblot analysis); upper panel: thermal treatment only; lower panel: thermal treatment with MB; (**b**) MB (5–20 μM, 1 h pretreatment) effects on celastrol-induced (1 μM, 24 h) upregulation of Hsp70 and Hsp27 protein levels (immunoblot analysis); (**c**–**e**) MB-modulation (10 μM, 1 h pretreatment) of celastrol-induced (1 μM, 24 h) heat shock response gene expression (*HSPA1A*, *HSPB1*, *HSPD1*) analyzed at the mRNA level using quantitative RT-PCR.

After demonstrating that MB exposure downregulates protein levels of Hsp70 and Hsp27 in human melanoma cells cultured under standard conditions, we examined the possibility that MB exposure could also attenuate cellular heat shock response gene expression elicited upon thermal or pharmacological challenge (Figure 4).

First, effects of MB (5 μM, 1 h pretreatment) on heat-induced (43 °C) upregulation of Hsp70 protein levels in A375 cells were assessed by Hsp70 immunoblot analysis (Figure 4a). Indeed, pronounced suppression of thermally induced expression of Hsp70 was observed in response to MB exposure. Next, we used the established small molecule heat shock inducer celastrol (1 μM, 24 h), an electrophilic quinone-methide-based natural product, for the pharmacological induction of heat shock response gene expression in A375 melanoma cells [47–49]. As expected, celastrol exposure caused the pronounced upregulation of Hsp70 and Hsp27 at the protein (Figure 4b) and transcriptional level (*HSPA1A*, Figure 4C; *HSPB1*, Figure 4d).

MB treatment caused pronounced attenuation of celastrol-induced heat shock response gene expression (*HSPA1A*, *HSPB1*) in A375 cells, an effect observed at the protein (Figure 4b) and mRNA level (Figure 4c,d). In contrast, in response to celastrol exposure cellular levels of Hsp60 (encoded by *HSPD1*) remained unchanged at the protein (data not shown) and mRNA level (Figure 4e), consistent with the known irresponsiveness of Hsp60 to regulation by heat shock induction.

### 2.4. Methylene Blue Attenuates Geldanamycin-Induced *HSPA1A* Upregulation and Sensitizes Human A375 Metastatic Melanoma Cells to Geldanamycin-Induced Apoptosis

Guided by our observation that MB treatment can attenuate stress-induced heat shock response gene expression in cultured metastatic melanoma cells, we tested feasibility of using MB to suppress upregulation of Hsp70 expression in a pharmacological context potentially relevant to chemotherapeutic elimination of cancer cells.

First, we examined the possibility that MB cotreatment at non-lethal concentrations can enhance apoptogenicity of established cancer chemotherapeutics known to be antagonized by Hsp70 expression [50,51].

**Figure 5:**
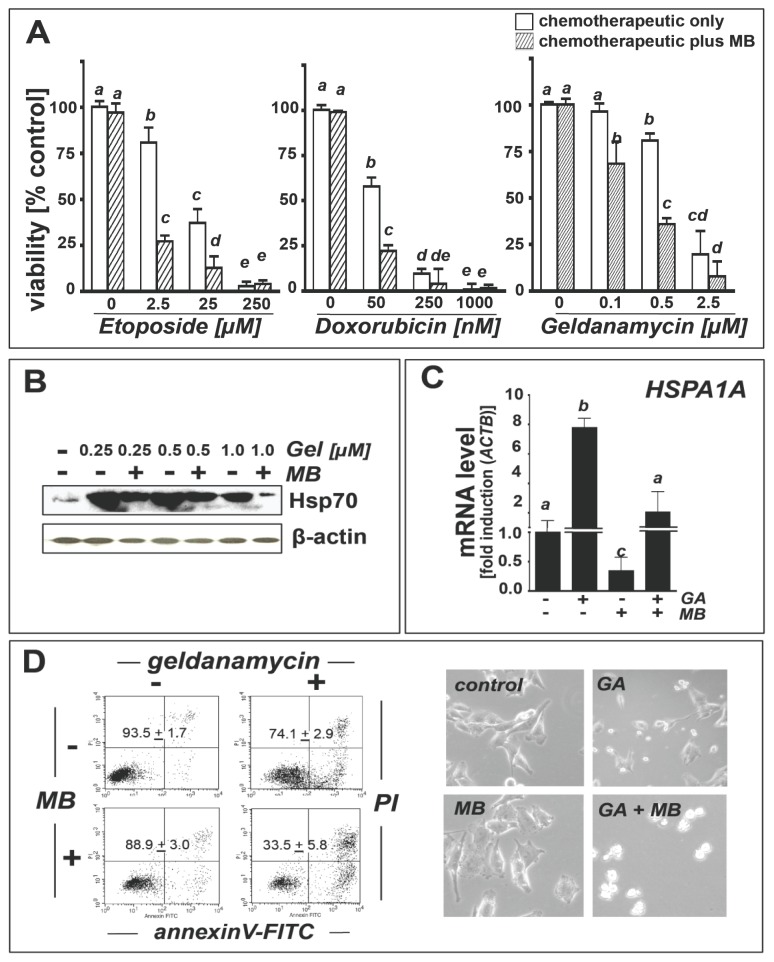
Methylene blue sensitizes A375 melanoma cells to etoposide-, doxorubicin-, and geldanamycin-induced cell death and attenuates geldanamycin-induced *HSPA1A* upregulation. (**a**) MB-induced (10 μM cotreatment; 24 h) sensitization to etoposide, doxorubicin, and geldanamycin (GA) cytotoxicity (CellTiter-Glo™ luminescence analysis); (**b**) MB attenuation (10 μM; 1h pretreatment) of GA-induced (≤1.0 μM, 24 h) Hsp70 upregulation (immunoblot analysis); (**c**) MB attenuation (10 μM; 1 h pretreatment) of GA-induced (0.5 μM, 24 h) *HSPA1A* mRNA upregulation; (**d**) MB-sensitization (10 μM; 1 h pretreatment) to GA-induced cell death (0.5 μM, 24 h) (flow cytometric analysis; left panels). Numbers indicate viable (AV-negative, PI-negative) in percent of total gated cells (mean ± SD, *n* = 3); right panels: Representative light microscopy pictures (24 h).

Indeed, in A375 melanoma cells, cytotoxicity of etoposide and doxorubicin was significantly potentiated by MB cotreatment (Figure 5a). Potentiation of etoposide cytotoxicity was most pronounced at 2.5 μM concentration where MB cotreatment caused a threefold reduction of the viable melanoma cell fraction, an extent of cell death equal to that induced by 25 μM etoposide if used as single agent (Figure 5a, left panel). Similarly, a moderate MB-induced potentiation of doxorubicin (50 nM) cytotoxicity (Figure 5a, middle panel) was observed.

It has been shown earlier that the apoptogenic efficacy of geldanamycin, a cytotoxic Hsp90 antagonist and investigational cancer chemotherapeutic, is compromised as a result of counter-regulatory upregulation of Hsp70 expression, an established cytoprotective mechanism that depends on heat shock factor (HSF1) transcriptional activation downstream of Hsp90 inhibition [5,7,52].

In order to specifically test the hypothesis that MB-induced Hsp70-antagonism may cause the chemosensitization of melanoma cells towards geldanamycin-induced apoptosis, we therefore examined modulation of Hsp70 expression at the protein (Figure 5b) and mRNA (*HSPA1A*) levels (Figure 5c). We also assessed induction of apoptosis in A375 metastatic melanoma cells exposed to the single or combined action of MB and geldanamycin (Figure 5a,d). Indeed, geldanamycin (0.25–1 μM) caused pronounced Hsp70 upregulation at the protein (Figure 5b) and mRNA level (Figure 5c). MB co-treatment strongly attenuated geldanamycin-induced Hsp70 upregulation, an effect observable at the protein (Figure 5b) and mRNA level (Figure 5c). Geldanamycin-induction of Hsp70 protein levels was most significant at concentrations <1 μM and was less pronounced at 1 μM, an observation consistent with the pronounced cytotoxicity of the agent that might impair Hsp70 expression at higher doses.

Geldanamycin-based chemotherapeutic intervention has shown only limited efficacy in melanoma models [53]. We therefore examined feasibility of achieving chemosensitization towards geldanamycin-induced apoptosis by MB co-administered at non-cytotoxic concentrations (Figure 5a, right panel and Figure 5d, left panels). Significant MB-potentiation of geldanamycin cytotoxicity became apparent when MB (10 μM) combined with geldanamycin (0.5 μM) caused an extent of cell death almost equal to that induced by a fivefold higher geldanamycin concentration (2.5 μM) if used as a single agent (Figure 5a, right panel). Flow cytometric analysis of annexinV-propidium iodide stained A375 cells after exposure to the single or combined action of geldanamycin (0.5 μM, 24 h) and MB confirmed a pronounced sensitization to geldanamycin-induced cell death achieved by MB co-administration (Figure 5d, left panels; geldanamycin only: 74.1% + 2.9% viable cells; geldanamycin/MB co-treatment: 33.5% ± 5.8% viable cells; mean ± S.D.; *n* = 3), an effect that was also observable by light microscopy (Figure 5d, right panels).

Taken together, our data demonstrate feasibility of achieving chemosensitization of cultured melanoma cells to the apoptogenic acitivity of chemotherapeutics including etoposide, doxorubicin, and geldanamycin. MB-attenuation of geldanamycin-induced Hsp70 upregulation suggests that chemosensitization may in part result from MB-dependent suppression of heat shock response gene expression, a hypothesis to be substantiated further by future experimentation.

## 3. Experimental Section

### 3.1. Chemicals

All chemicals were purchased from Sigma Chemical Co. (St. Louis, MO, USA).

### 3.2. Cell Culture

A375 and G-361 human metastatic melanoma cells from ATCC (Manassas, VA, USA) were cultured in RPMI medium containing 10% FBS and 2 mM l-glutamine or McCoy’s 5a medium containing 10% FBS, respectively. Cells were maintained at 37 °C in 5% CO_2_, 95% air in a humidified incubator. For thermal induction of heat shock, cells were first exposed to 43 °C (0–120 min exposure) and then maintained for 6 h at 37 °C (5% CO_2_, 95% air).

### 3.3. Human Stress and Toxicity Pathfinder™ RT^2^ Profiler™ PCR Expression Array

After pharmacological exposure, total cellular RNA (3 × 10^6^ cells) was prepared according to a standard procedure using the RNeasy kit (Qiagen, Valencia, CA, USA). Reverse transcription was performed using the RT^2^ First Strand kit (SuperArray, Frederick, MD, USA) and 5 μg total RNA as described previously [25]. The RT^2^ Human Stress and Toxicity Pathfinder™ PCR Expression Array (SuperArray) profiling the expression of 84 stress-related genes was run using the following PCR conditions: 95 °C for 10 min, followed by 40 cycles of 95 °C for 15 s alternating with 60 °C for 1 min (Applied Biosystems 7000 SDS, Carlsbad, CA, USA). Gene-specific product was normalized to GAPDH and quantified using the comparative (ΔΔC_t_) Ct method as described in the ABI Prism 7000 sequence detection system user guide as published earlier [25]. Expression values were averaged across three independent array experiments, and standard deviation was calculated for graphing.

### 3.4. *HSPA1A*, *HSPB1*, *HSPD1*, *HSP90AA1* Expression Analysis by Real Time RT-PCR

For expression analysis of selected genes by real time RT-PCR, total cellular RNA (3 × 10^6^ cells) was prepared using the RNEasy kit from Qiagen (Valencia, CA, USA). Reverse transcription was performed using TaqMan Reverse Transcription Reagents (Roche Molecular Systems, Branchburg, NJ, USA) and 200 ng of total RNA in a 50 μL reaction. Reverse transcription was primed with random hexamers and incubated at 25 °C for 10 min followed by 48 °C for 30 min, 95 °C for 5 min, and a chill at 4 °C. Each PCR reaction consisted of 3.75 μL of cDNA added to 12.5 μL of TaqMan Universal PCR Master Mix (Roche Molecular Systems, Pleasanton, CA, USA), 1.25 μL of gene-specific primer/probe mix (Assays-by-Design; Applied Biosystems, Foster City, CA, USA) and 7.5 μL of PCR water. PCR conditions were: 95 °C for 10 min, followed by 40 cycles of 95 °C for 15 s, alternating with 60 °C for 1 min using an Applied Biosystems 7000 SDS and Applied Biosystems’ Assays On Demand primers specific to, *HSPA1A* (assay ID Hs00359163_s1), *HSPB1* (assay ID Hs00356629_g1), *HSPD1* (assay ID Hs01866140_g1), *HSP90AA1* (assay ID Hs00743767_sH), and *ACTB* (assay ID Hs01060665_g1). Gene-specific product was normalized to *ACTB* and quantified using the comparative (ΔΔC_t_) Ct method as described before [25,40].

### 3.5. Immunoblot Analysis

Sample preparation, SDS-PAGE, transfer to nitrocellulose, and development occurred as described earlier [25,40,54]. Gel percentage was 12%. Antibodies were purchased from the following manufacturers: Cell Signaling Technology (Danvers, MA, USA): anti-p21 rabbit monoclonal (12D1); Enzo Life Sciences (Farmingdale, NY, USA): anti-Hsp70 mouse monoclonal (C92F3A-5, SPA-810; this antibody recognizes inducible Hsp70, also called Hsp72 or Hsp70-1, but does not react with constitutively expressed Hsc70); anti-Hsp60 mouse monoclonal (Mab11-13; SPA-829) anti-Hsp90 rabbit polyclonal (SPA-836); anti-Hsp27 rabbit polyclonal (SPA-803).

The following secondary antibodies were used: HRP-conjugated goat anti-rabbit antibody or HRP-conjugated goat anti-mouse antibody (Jackson Immunological Research, West Grove, PA, USA). Equal protein loading was examined by β-actin-detection using a mouse anti-actin monoclonal antibody (Sigma, St. Louis, MO, USA).

### 3.6. GM-CSF ELISA

The enzyme-linked immunosorbent assay for colorimetric GM-CSF detection in culture medium of MB-exposed (1–20 μM) A375 and G361 melanoma cells (1 × 10^6^ cells) was performed in 96 well format using recombinant protein standard according to kit instructions (Human GM-CSF ELISA Kit; Thermo Scientific, Ann Arbor, MI, USA). Absorbance (450 and 550 nm) was determined on a microtiter plate reader (Versamax, Molecular Devices, Sunnyvale, CA, USA). Data represent the average of three independent experiments.

### 3.7. Cell Death Analysis

Viability and induction of cell death (early and late apoptosis/necrosis) were examined by annexin-V-FITC (AV)/propidium iodide (PI) dual staining of cells followed by flow cytometric analysis as published previously [24]. Cells (100,000) were seeded on 35 mm dishes and received drug treatment 24 h later. Cells were harvested at various time points after treatment and cell staining was performed using an apoptosis detection kit according to the manufacturer’s specifications (APO-AF, Sigma, St. Loius, MO, USA). For chemosensitization experiments, the CellTiter-Glo™ assay (Promega) based on luciferase-dependent luminescent detection was performed in homogeneous 96 well-format according to the manufacturer’s instruction [41].

### 3.8. Detection of Intracellular Oxidative Stress by Flow Cytometric Analysis

Induction of intracellular oxidative stress by test compound was analyzed by flow cytometry using 2′,7′-dichlorodihydrofluorescein diacetate (DCFH-DA) as a sensitive non-fluorescent precursor dye according to a published standard procedure [24].

### 3.9. Determination of Reduced Cellular Glutathione Content

Intracellular reduced glutathione was measured using the GSH-Glo Glutathione assay kit (Promega; San Luis Obispo, CA, USA) as described recently [55]. Cells were seeded at 100,000 cells/dish on 35 mm dishes. After 24 h, cells were treated with test compound. At selected time points after addition of test compound, cells were harvested by trypsinization and then counted using a Coulter counter. Cells were washed in PBS, and 10,000 cells/well (50 μL) were transferred onto a 96-well plate. GSH-Glo reagent (50 μL) containing luciferin-NT and glutathione-*S*-transferase was then added followed by 30 min incubation. After addition of luciferin detection reagent to each well (100 μL) and 15 min incubation luminescence reading was performed using a BioTek Synergy 2 Reader (BioTek, Winooski, VT, USA). Data are normalized to GSH content in untreated cells and expressed as means ± SD (*n* = 3).

### 3.10. Statistical Analysis

Unless indicated differently, the results are presented as mean ± S.D. of at least three independent experiments, and data were analyzed employing *one-way* analysis of variance (*ANOVA*) with Tukey’s *post hoc* test using the Prism 4.0 software (version 4.0a, GraphPad Software, Inc., San Diego, CA, USA, 2003). Differences were considered significant at *p* < 0.05. Means without a common letter differ (*p* < 0.05).

## 4. Conclusions

Small molecule heat shock response antagonists are an emerging class of targeted chemotherapeutics for induction of cancer cell apoptosis [7–10,56,57]. Among a collection of 3,7-diaminophenothiazinium compounds we have identified the clinically used redox-active phenothiazine drug MB as a negative modulator of thermally and pharmacologically induced heat shock response gene expression in cultured human metastatic melanoma cells. Importantly, MB treatment attenuates geldanamycin-induced Hsp70 upregulation, and, when combined with other chemotherapeutics, MB-treatment causes significant chemosensitization of cultured melanoma cells. However, structure-activity and molecular mechanism underlying MB-induced heat shock response attenuation targeting expression of Hsp27 (*HSPB1*) and inducible Hsp70 (*HSPA1A*) remain undefined at this point and must be the subject of more detailed follow up studies. Indeed, MB is a promiscuous pharmacological agent known to interact with multiple cellular targets (including guanylate cyclase, nitric oxide synthase, monoamine oxidase, NQO1, Hsp70-ATPase, components of the mitochondrial respiratory chain, and the proteasome) through redox-directed and redox-independent mechanisms that may also be involved in MB-modulation of heat shock response gene expression [24,28–38,44]. The molecular mechanism and functional implications of MB-induced upregulation of GM-CSF expression as observed by us for the first time remain to be explored in the context of its potential usefulness for immunostimulatory antimelanoma intervention [41,42]. Moreover, irrespective of the molecular mechanisms involved, it remains to be seen if heat shock response gene expression observed here in cultured melanoma cells can also be attenuated in melanoma tumor tissue upon systemic administration of MB, a question to be assessed *in vivo* in relevant animal models of the disease.

Repurposing approved and abandoned non-oncological drugs as an alternative developmental strategy for the identification of anticancer therapeutics has recently attracted considerable attention [58,59]. Based on a defined pharmacokinetic and toxicological profile and its established clinical use as an infusional and oral drug for non-oncological indications in human patients [28], our data suggest feasibility of repurposing MB as an investigative heat shock response antagonist for the chemosensitization of human cancer cells.
